# Maternal sepsis: a Scottish population-based case–control study

**DOI:** 10.1111/j.1471-0528.2011.03239.x

**Published:** 2012-01-18

**Authors:** CD Acosta, S Bhattacharya, D Tuffnell, JJ Kurinczuk, M Knight

**Affiliations:** a National Perinatal Epidemiology Unit, University of OxfordOxford; b Obstetrics and Gynaecology, Division of Applied Health Sciences, University of AberdeenScotland; c Bradford Royal InfirmaryBradford, UK

**Keywords:** Maternal sepsis, obesity, operative vaginal delivery

## Abstract

**Objective:**

To describe the risk of maternal sepsis associated with obesity and other understudied risk factors such as operative vaginal delivery.

**Design:**

Population-based, case–control study.

**Setting:**

North NHS region of Scotland.

**Population:**

All cases of pregnant, intrapartum and postpartum women with International Classification of Disease-9 codes for sepsis or severe sepsis recorded in the Aberdeen Maternal and Neonatal Databank (AMND) from 1986 to 2009. Four controls per case selected from the AMND were frequency matched on year-of-delivery.

**Methods:**

Cases and controls were compared; significant variables from univariable regression were adjusted in a multivariable logistic regression model.

**Main outcome measures:**

Dependent variables were uncomplicated sepsis or severe (‘near-miss’) sepsis. Independent variables were demographic, medical and clinical delivery characteristics. Unadjusted and adjusted odds ratios (OR) with 95% confidence intervals (95% CI) are reported.

**Results:**

Controlling for mode of delivery and demographic and clinical factors, obese women had twice the odds of uncomplicated sepsis (OR 2.12; 95% CI 1.14–3.89) compared with women of normal weight. Age <25 years (OR 5.15; 95% CI 2.43–10.90) and operative vaginal delivery (OR 2.20; 95% CI 1.02–4.87) were also significant predictors of sepsis. Known risk factors for maternal sepsis were also significant in this study (OR for uncomplicated and severe sepsis respectively): multiparty (OR 6.29, 12.04), anaemia (OR 3.43, 18.49), labour induction (OR 3.92 severe only), caesarean section (OR 3.23, 13.35), and preterm birth (OR 2.46 uncomplicated only).

**Conclusions:**

Obesity, operative vaginal delivery and age <25 years are significant risk factors for sepsis and should be considered in clinical obstetric care.

## Introduction

Severe sepsis is a potentially life-threatening condition that is characterised by systemic inflammatory response syndrome with infection, organ dysfunction, hypoperfusion and hypotension.^1^ If untreated, sepsis can rapidly progress along a continuum of severity to septicaemic shock and eventually death. Pregnant and postpartum women represent a particularly vulnerable population for developing severe sepsis because of changing physiology, biochemistry and immune response,^2^ as well as heightened susceptibility to wound infection during delivery.

Although death as a result of pregnancy-related sepsis is uncommon in the UK and other high-income countries, mortality rates have more than doubled over the last two decades in the UK^3^ and have also increased in other European countries.^4^ In the late 1980s the maternal mortality rate from sepsis in the UK was 0.4/100 000 maternities, whereas in the period from 2006 to 2008 the maternal mortality rate increased to 1.13/100 000.^3^ This rate places sepsis as the leading cause of direct maternal death, surpassing that of hypertensive disorders of pregnancy.^3^ In addition, each fatality represents a much larger incidence of morbidity and ‘near miss’ events during the perinatal and puerperal periods. In the Netherlands, sepsis accounted for 8.1% of all Dutch obstetric intensive-care unit admissions.^5^ Concurrently, a recent body of evidence has indicated increasing incidence and severity of sepsis in the general populations of both the USA and Europe,^6^–^9^ accounting for 10–64% of all intensive-care unit admissions.^10^–^12^ Given the recent increase in maternal deaths and an increase in morbidity in the general population caused by sepsis, an understanding of the risk factors for sepsis in pregnant and postpartum women is needed to better target potential points of clinical intervention.^13^,^14^

There are several well-established risk factors for maternal sepsis including caesarean section^15^–^18^ and anaemia.^17^,^19^ However, the risk of maternal sepsis being potentially associated with obesity has not been well described, although obesity has been implicated in poor wound healing (primarily in relation to caesarean section),^20^–^24^ genitourinary infection^20^ and uterine infection^21^ in the obstetric population. The LEMMoN study from the Netherlands recently estimated the relative risk of maternal sepsis associated with being overweight (body mass index ≥25 and <30) to be 1.6 (95% CI 0.9–2.8), although this was not statistically significant.^18^ In the UK, as in other developed countries, obesity rates including women of reproductive age have been increasing rapidly.^25^,^26^ In the mid-2000s, approximately 20% of pregnant women in the UK were obese.^27^,^28^ From 2003 to 2005, 33% of mothers who died directly from sepsis in the UK were obese, and 48% had undergone a caesarean section, all of whom were either overweight or obese.^29^

The aim of this study, therefore, was to describe the risk of sepsis or severe sepsis associated with obesity (body mass index ≥30) among pregnant, intrapartum and immediately postpartum women. A secondary aim was to describe understudied risk factors such as operative vaginal delivery, as well as known risk factors not previously reported in the UK. Our hypotheses were that (i) controlling for other clinical factors including mode of delivery, the risk of maternal sepsis would be higher for obese women, and that (ii) based on findings from other countries, operative vaginal delivery would be a significant risk factor for maternal sepsis.

## Methods

This study was conducted using data from the University of Aberdeen, Aberdeen Maternity Hospital, a tertiary-care maternity hospital for the NHS North of Scotland region. The hospital is the only tertiary referral hospital in the area, serves a large and well-defined geographical region and has approximately 5000 births per year. All pregnancy-related events have been recorded in the Aberdeen Maternal and Neonatal Databank (AMND) since 1950. Data entry, coding protocols and consistency rates for internal validation and valid ranges of measurable variables are described in previous studies.^30^–^32^

### Study design and outcome variables

This was an anonymised case–control study of all maternal sepsis among antepartum, intrapartum and postpartum women recorded in the AMND between 1986 and 2009. Data were analysed using a case–control design because of the relative rarity of maternal sepsis in the UK, and because there are multiple known risk factors for maternal sepsis. All cases were identified as those with an International Classification of Disease ninth revision (ICD-9) sepsis code: 038.0–038.9 (septicaemia), 634.0–639.0 (sepsis following abortion), 670.2 (puerperal sepsis) and 785.5 (septic shock). Severe (‘near-miss’) sepsis cases were identified as those with an ICD-9 code of septic shock, or according to previously validated criteria defined by Martin et al.,^6^ which were those with an additional ICD-9 code for acute organ dysfunction associated with sepsis. All other cases of sepsis are referred to as ‘uncomplicated’. All cases with an ICD-9 code for sepsis had a clinical diagnosis for systemic inflammatory response syndrome in addition to a culture-confirmed diagnosis of infection. Four controls per case selected from the AMND who did not have an ICD-9 code for sepsis were frequency matched on year of delivery.

Research ethics committee approval for use of anonymised data was not required. Approval of the research protocol was obtained from the Steering Committee of the AMND before data extraction.

### Sample size and statistical analyses

The sample size of this study was limited by the population incidence of maternal sepsis, thereby minimising the possibility of selection bias. The predictor variable of primary interest was obesity. Therefore, with 103 cases (all cases occurring in the population over the study period) and four controls per case, and assuming a prevalence of exposure to obesity of 19%,^25^ the study had 90% power at *P* < 0.05 (two-sided) to detect a statistically significant odds ratio (OR) of 2.3 or greater; this OR corresponds to the odds reported in the most recent European study of intra-amniotic infection among obese pregnant women.^33^

Frequencies of demographic and clinical variables were tabulated for uncomplicated sepsis and severe sepsis case groups and compared with controls using chi-square tests for categorical variables and a Student's *t* test or Wilcoxon rank sum test as appropriate for continuous variables. Univariable logistic regression analyses were carried out to initially identify demographic and clinical risk factors for uncomplicated maternal sepsis and severe sepsis; all *P*-values were unadjusted and two-sided, and a *P*-value of <0.05 was considered statistically significant. Significant variables from univariable regression, or those factors that were plausible confounders were included in a multivariable logistic regression model using a stepwise method. Potential confounding factors that were not significant at *P* < 0.05 in the initial multivariable regression (blood loss of ≥500 ml, pre-eclampsia and previous miscarriage) were removed from the final model. Interactions between demographic and clinical variables were assessed using likelihood ratio tests with a significance level of *P* < 0.01; no interactions were identified in the final adjusted model. Only two cases occurred antenatally and removal of these cases did not significantly change the regression results, so the antenatal cases were retained in the final regression models. Multivariable regression results were adjusted for calendar time and all other factors in the model. Results are reported as unadjusted and adjusted OR with 95% confidence intervals (95% CI). Stata statistical software 11 (StataCorp, College Station, TX, USA) was used for all analyses.

## Results

**Figure 1:**
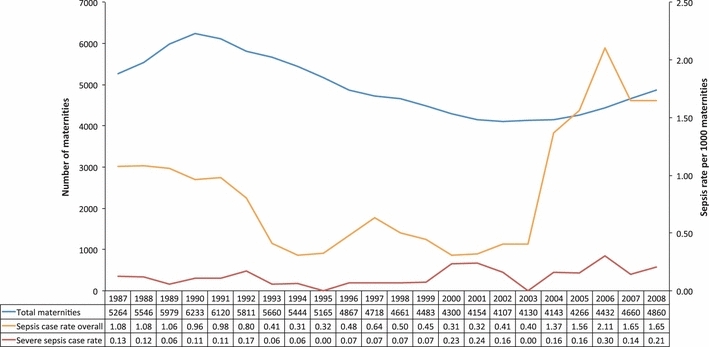
Incidence of all sepsis and severe sepsis cases per 1,000 maternities from 1986-2009 in Aberdeen (three-year rolling averages).

The total study population comprised 89 cases of uncomplicated maternal sepsis, 14 cases of severe maternal sepsis and 412 control women. The majority of all cases occurred postpartum and before hospital discharge; 98.9% (*n* = 88) of women with uncomplicated sepsis and 92.9% (*n* = 13) of women with severe sepsis. One woman had uncomplicated sepsis antepartum, and one women with severe sepsis was diagnosed with an infection during delivery, which later progressed to septic shock postpartum. Sepsis case rates for the 23-year study period are shown in Figure 1. Although rates of severe sepsis remained relatively constant over the study period, rates of sepsis overall have increased significantly since 2003 (*P* = 0.002) compared with the previous two decades. Point estimates for 3-year averages of overall sepsis case rates for the periods 1987–89, 1997–99 and 2007–09 were: 1.08 per 1000 maternities (95% CI 0.64–1.71), 0.43 (95% CI 0.16–0.93) and 1.65 (95% CI 1.05–2.45) respectively. Point estimates for 3-year averages of severe sepsis for the same periods were: 0.12 per 1000 maternities (95% CI 0.01–0.43), 0.07 (95% CI 0.00–0.40) and 0.21 (95% CI 0.02–0.50).

The distribution of several demographic and medical characteristics differed significantly between the three groups (Table 1). The proportion of women from black or other minority ethnic groups was greater among women with sepsis: 5.7% of uncomplicated sepsis and 21.4% of severe sepsis, compared with 0% of controls. The average age of women with uncomplicated sepsis and severe sepsis was younger than that of controls: mean age 28.2 years (SD 5.6) and 27.4 years (SD 6.2), respectively, compared with 31.3 years (SD 4.5). A larger proportion of women with sepsis were multiparous and also had a previous miscarriage. Additionally, the proportion of mothers with anaemia was progressively larger among women with uncomplicated sepsis and severe sepsis: 67.4% and 92.9% respectively compared with 37.6% of control women. Although the rate of obesity was significantly greater among women with sepsis, obesity among severe sepsis cases was similar to that of controls. Gestation at booking, marital status and diabetes did not differ significantly between groups. One obese woman among the women with sepsis and one obese woman among the controls had type 1 diabetes.

**Table 1:** Demographic and medical characteristics of cases and controls (1986–2009)

	Uncomplicated sepsis	Severe sepsis	Controls	*P*-value*
				
	*n* = 89	*n* = 14	*n* = 412	
Ethnic group**				
White	82 (94.3)	11 (78.6)	408 (100.0)	<0.001
Black	2 (2.3)	1 (7.1)	–	
Asian	3 (3.5)	2 (14.3)	–	
**Age, years, mean (SD)**	28.2 (5.6)	27.4 (6.2)	31.3 (4.5)	<0.001
**Parity**				
0	25 (28.1)	5 (35.7)	178 (43.2)	0.009
1	39 (43.8)	5 (35.7)	179 (43.5)	0.89
≥2	25 (28.1)	4 (28.6)	55 (13.3)	<0.001
**First booking**				
First trimester	77 (86.5)	14 (100.0)	316 (82.7)	0.53
Second or third trimester	12 (13.5)	0 (0.0)	66 (17.3)	
**Marital status**				
Married	59 (66.3)	11 (78.6)	297 (72.1)	0.41
Single supported	17 (19.1)	2 (14.3)	73 (17.7)	0.86
Single unsupported	10 (11.2)	1 (7.1)	33 (8.0)	0.39
Divorced or separated	3 (3.4)	0 (0.0)	9 (2.1)	0.1
Body mass index, median (interquartile range)**	26 (23–30)	24 (22–25)	24 (22–28)	0.05
**Obese**	32 (36.0)	2 (14.3)	78 (18.9)	0.002
**Anaemia**	60 (67.4)	13 (92.9)	155 (37.6)	<0.001
**Diabetes (type 1)**	2 (2.3)	0 (0.0)	3 (0.7)	0.26
**Any previous miscarriage**	6 (6.7)	2 (14.3)	12 (2.9)	0.023

Note categories are mutually exclusive.

Values are numbers (%) of women unless otherwise stated.

* Difference in distribution between all cases and controls; chi-square test; Fisher's exact test for fewer than five observations; Student's *t* test for mean age; rank sum test for median body mass index.

** Among those reported.

Several delivery characteristics also differed significantly across the three groups (Table 2). A larger proportion of women with severe sepsis had prelabour rupture of membranes and an induced labour compared with controls. More women with uncomplicated and severe sepsis had caesarean sections: 48.3% and 64.3%, respectively, compared with 25.3% of controls. A larger proportion of women with severe sepsis also had a manual removal of the placenta (21.4% versus 4.7% of controls, *P* = 0.04) and lost ≥500 ml blood (71.4% versus 23.9% of controls, *P* = 0.02) during delivery. The rate of severe pre-eclampsia, preterm birth and babies admitted to the neonatal intensive-care unit was also higher among women with sepsis.

**Table 2:** Clinical delivery characteristics of cases and controls

	Uncomplicated sepsis	Severe sepsis	Controls	*P*-value*
				
	*n* = 89	*n* = 14	*n* = 412	
Premature rupture of membranes (>18 hours before labour onset)**	5 (7.6)	2 (25.0)	38 (10.2)	0.84
Type of membrane rupture**				
Artificial	32 (43.8)	3 (27.3)	190 (49.5)	0.19
Spontaneous	41 (56.2)	8 (72.7)	194 (50.5)	0.19
**Type of labour**				
Spontaneous	46 (51.7)	3 (21.4)	251 (60.9)	0.014
Induced	31 (34.8)	9 (64.3)	139 (33.7)	0.33
Elective caesarean	12 (13.5)	2 (14.3)	22 (5.3)	0.003
Prolonged labour (stages 1 and 2 >12 hours)*	28 (41.2)	5 (45.5)	133 (35.0)	0.25
**Mode of delivery**				
Spontaneous vaginal	29 (32.6) (63.0)***	2 (14.3) (40.0)***	202 (49.2) (16.0)***	0.001
Operative vaginal****	17 (19.1) (37.0)***	3 (21.4) (60.0)***	105 (25.5) (34.2)***	0.2
caesarean section	43 (48.3)	9 (64.3)	104 (25.3)	<0.001
**Type of placental delivery**				
Controlled cord traction	83 (93.3)	10 (71.4)	380 (93.4)	0.28
Maternal effort	1 (1.1)	1 (7.1)	8 (2.0)	0.99
Manual removal	5 (5.6)	3 (21.4)	19 (4.7)	0.21
**Blood loss at delivery (ml)**				
<200	15 (16.9)	3 (21.4)	110 (26.8)	0.5
≥200 <500	40 (44.9)	1 (7.1)	202 (49.3)	0.086
≥500	34 (38.2)	10 (71.4)	98 (23.9)	<0.001
**Perineal wound**	30 (33.7) (65.2)***	2 (14.3) (40.0)***	253 (61.4) (82.4)***	0.42
Antibiotics during pregnancy or delivery*****	37 (41.6)	3 (21.4)	176 (42.7)	0.48
**Complications in current pregnancy**				
Severe pre-eclampsia	16 (18.0)	3 (21.4)	31 (7.5)	0.001
Haemorrhage, placental abruption or placenta praevia	12 (13.5)	2 (14.3)	43 (10.4)	0.55
Preterm birth	17 (19.1)	3 (21.4)	30 (7.3)	<0.001
Baby admitted to neonatal intensive-care unit**	27 (42.2)	4 (44.4)	62 (21.6)	<0.001

Note categories are mutually exclusive.

Values are numbers (%) of women.

* Difference in distribution between all cases and controls; chi-square test; Fisher's exact test for fewer than five observations.

** Among those reported.

*** Proporion of all vaginal delivery.

**** Operative vaginal delivery includes the use of: forceps, Kielland forceps, vacuum extraction, assisted breech, and breech extraction.

***** Antibiotics administered before discharge from the labour ward.

Over the 23-year study period, the proportion of spontaneous vaginal deliveries decreased while the proportion of operative vaginal deliveries and caesarean sections increased significantly (*P* < 0.001; *P* = 0.009; *P* < 0.001, respectively). Despite these trends, sepsis case rates remained predominantly the highest among women who had a caesarean section throughout the study period, although rates increased in all modes from 2003 onwards. Antibiotic usage (before discharge from the labour ward) in the overall study population increased steadily from 1986 to 1996 (*P* < 0.001) and plateaued from 1997 to 2009. Although there was no significant difference between cases and controls in the total proportion of antibiotic usage across all modes of delivery (Table 2), among the women with sepsis who had a caesarean section, 38.5% received antibiotics during pregnancy or delivery, which was significantly lower than the 70.2% of control women who had a caesarean section and received antibiotics (*P* < 0.001). In addition, antibiotic usage among control women who had a caesarean section increased significantly over time (*P* = 0.003), whereas antibiotic usage among cases who had a caesarean section did not increase over time. There was no significant difference in antibiotic usage among women in the case and control groups who had a manual placenta removal or an operative vaginal delivery.

After adjusting for changes over time and other factors in each model respectively, factors significantly associated with both uncomplicated sepsis and severe sepsis were: younger age, multiparity, anaemia, operative vaginal delivery and caesarean section (compared with spontaneous vaginal delivery) (Table 3). Obesity was significantly associated with uncomplicated sepsis; however, this association was not present with severe sepsis. Additionally, operative vaginal delivery and preterm birth were associated with uncomplicated sepsis, whereas induced labour was associated with severe sepsis.

**Table 3:** Unadjusted and adjusted odds ratios for factors associated with uncomplicated and severe sepsis

	Uncomplicated sepsis				Severe sepsis			
		
	Unadjusted		Adjusted		Unadjusted		Adjusted	
				
	OR	95% CI	OR	95% CI	OR	95% CI	OR	95% CI
**Demographic charateristics**								
Age tertiles								
<25 years	4.94	2.64–9.22	5.15	2.43–10.90	5.10	1.32–19.78	10.17	1.86–55.52
25–34 years	2.88	1.68–4.93	2.56	1.37–4.78	2.63	0.74–9.27	3.26	0.61–17.41
>34 years	1*		1*		1*		1*	
Parity								
0	1*		1*		1*		1*	
1	7.13	3.90–13.01	7.44	3.64–15.23	4.47	1.10–18.15	6.31	0.94–42.55
≥2	3.54	1.91–6.56	6.29	2.88–13.77	4.15	1.17–14.72	12.04	2.07–69.90
Late booking (second or third trimester)	0.96	0.49–1.88			–	–		
Marital status								
Married	1*				1*			
Single supported	1.17	0.65–2.13			0.74	0.16–3.41		
Single unsupported	1.52	0.71–3.26			0.82	0.10–6.54		
Divorced or separated	5.03	0.99–25.55			–			
**Medical characteristics**								
Obese	2.4	1.46–3.96	2.12	1.14–3.89	0.71	0.16–3.25		
Smoked during pregnancy	1.46	0.86–2.48			0.30	0.04–2.36		
Anaemia	3.43	2.11–5.58	3.44	1.93–6.13	21.55	2.79–166.38	1.97–173.14	
Diabetes	3.13	0.52–19.04			–	–		
Any previous miscarriage	2.41	0.88–6.60			5.56	1.12–27.61		
**Clinical delivery characteristics**								
Premature rupture of membranes	0.72	0.27–1.90			2.92	0.57–14.99		
Type of membrane rupture								
Artificial	1*				1*			
Spontaneous	1.25	0.76–2.08			2.61	0.68–9.99		
Induced labour	1.05	0.65–1.70			3.54	1.16–10.75	3.92	1.02–15.35
Prolonged labour	1.30	0.77–2.20			1.55	0.46–5.17		
Type of delivery								
Spontaneous vaginal	1*		1*		1*		1*	
Operative vaginal**	1.13	0.59–2.15	2.20	1.02–4.87	2.89	0.47–17.54	6.39	0.72–56.46
Caesarean section	2.88	1.70–4.88	3.23	1.65–6.34	8.74	1.85–41.19	13.35	2.08–85.68
Manual placenta removal	1.22	0.44–3.35			2.16	0.26–17.68		
≥500 ml blood loss at delivery	1.97	1.21–3.19			7.96	2.44–25.94		
Perineal wound	0.32	0.20–0.52			0.10	0.02–0.47		
Antibiotics during pregnancy or delivery***	0.95	0.60–1.52			0.37	0.10–1.33		
Severe pre-eclampsia	2.69	1.40–5.18			3.35	0.89–12.65		
Haemorrhage, placental abruption, or placenta praevia	1.34	0.67–2.65			1.43	0.31–6.60		
Preterm birth	3.00	1.58–5.74	2.46	1.11–5.47	3.47	0.92–13.13		

Results adjusted for calendar time and for all factors listed in the table.

–Indicates no comparison group, i.e. zero incidence in either case or control group.

* Reference group.

** Operative vaginal delivery includes the use of: forceps, Kielland forceps, vacuum extraction, assisted breech, and breech extraction.

*** Antibiotics administered before discharge from the labour ward.

## Discussion

Sepsis is now the leading cause of direct maternal death in the UK,^3^ giving rise to an urgent need to describe the risk factors for obstetric sepsis morbidity. This population-based study reports specifically on the increased risk of maternal sepsis associated with obesity, controlling for mode of delivery. In addition, this investigation provides insight about several understudied risk factors for maternal sepsis including operative vaginal delivery and maternal age, while corroborating other well-known risk factors.

Over the study period there was a significant increase in the incidence of maternal sepsis from 2003 onwards, compared with the average decreasing trend seen in this population since 1986. There were no changes in diagnostic coding that would explain this increase, as all maternal events were recorded according to ICD-9 codes during the entire study period. One possible explanation for this increase is that 2003 was a peak year in the UK for β-haemolytic group A streptococcal (*Streptococcus pyogenes*) infection,^34^–^36^ which has traditionally been a major cause of maternal and puerperal sepsis morbidity and mortality.^3^,^37^ Interestingly, case rates increased across all modes of delivery, which could either indicate nosocomial or community-acquired infection. As microbiological data were not available for this study, further investigation is needed to assess the impact of group A streptococci as well as other causative organisms on maternal sepsis and possible routes of infection. The average decreasing trend in the incidence of all maternal sepsis from 1986 to 2003 may also be explained by group A streptococcal activity; 1988 was a peak year for scarlet fever and probably for invasive infection as well in the UK, with a subsequent decrease in following years. Increasing antibiotic usage may have also helped to precipitate the decreasing trend in maternal sepsis case rates during this period.

Although overall antibiotic usage increased over the study period and despite a recent move to offering antibiotics to all women undergoing a caesarean section as of 2004 under the UK National Institute for Health and Clinical Excellence (NICE) guidelines,^38^ fewer than half of all cases that had a caesarean section received antibiotics before discharge from the labour ward compared with the majority of control women who had a caesarean section.

In this study, there was sufficient power to confidently demonstrate associations between uncomplicated sepsis and high-prevalence predictive factors in multivariable regression. However, because of the low population prevalence of type 1 diabetes and manual placenta removal, there was insufficient power to exclude the role of chance in the association between these variables and the odds of sepsis. In addition, the sample size for women with severe sepsis was very small, which resulted in a large degree of uncertainty as shown by wide confidence intervals for most factors. The results of the analysis of severe sepsis, therefore, should be interpreted as exploratory; future studies in a larger population are needed to validate and elucidate the risk factors for severe sepsis.

It is generally known that obese pregnant women are at increased risk of wound infection with sepsis as a possible sequela. This increased risk is particularly evident following caesarean section,^22^–^24^,^39^, which obese women undergo much more frequently than women of normal weight.^20^,^21^,^40^ The extent to which obesity may independently predispose women to maternal sepsis, however, has not been well described. We found that after controlling for mode of delivery, obese women had twice the odds of developing uncomplicated sepsis compared with women of normal weight. This association was independent of mode of delivery. The degrees to which prediabetes, impaired glucose tolerance and gestational diabetes in association with obesity may play a role in the risk of sepsis, remain to be investigated.

An interesting finding from our study was that younger maternal age was a significant risk factor for sepsis and severe sepsis, which is contrary to previous studies. Older women are at increased risk for several pregnancy complications and studies on or including maternal sepsis have reported findings commensurate with this general trend.^18^,^41^ We found, however, that being younger than 25 years, which was younger than the mean age for either case group, was strongly predictive of sepsis. Women younger than 25 years had five times the odds of developing uncomplicated sepsis and ten times the odds of developing severe sepsis, compared with women older than 34 years. Despite the finding that younger maternal age was associated with all sepsis in this study, multiparity was also strongly associated with sepsis. This seeming confluence resulted from the fact that cases tended to be younger and multiparous, whereas controls tended to be older and primiparous. We speculate that this observation may replicate patterns of community-acquired infection, particularly group A streptococcal infections, but this cannot be confirmed using these data.

One understudied risk factor that emerged from our study was the association between operative vaginal delivery (forceps, vacuum extraction or assisted breech), and maternal sepsis. We found that women who had an operative delivery had over twice the odds of uncomplicated sepsis compared with women who had a spontaneous vaginal delivery. This finding is consistent with the few previous studies to have investigated this risk factor. In a Canadian population-based study, Liu et al.^42^ found that women delivered by vacuum extraction or forceps had an odds of 1.24 (95% CI 1.09–1.41) or 1.62 (95% CI 1.40–1.87) respectively, of major puerperal infection. Lydon-Rochelle et al.^43^ also found in a US population-based study that women who had an operative vaginal delivery were 20% more likely (relative risk 1.2) to be re-hospitalised within 60 days of delivery for uterine infection.

This study also corroborates several previously cited risk factors for maternal sepsis. In keeping with findings from Waterstone et al.,^44^ the only other study of severe sepsis morbidity in the UK, we found that multiparity, caesarean section and anaemia were all significant predictors of sepsis. These are well-known risk factors and have also been identified in other studies.^17^,^18^,^45^ As a result of the homogeneity of the control population, we were unable to evaluate ethnicity, a known risk factor for sepsis,^18^ in our regression model. However, given that there is very little ethnic variation in Aberdeen, it was striking that approximately 8% of cases were of non-white ethnic origin, whereas all controls were white.

Regarding anaemia, this condition can be caused by blood loss during delivery or by sepsis itself. Given that results of routine testing for antenatal anaemia are recorded in the AMND and the proportion of anaemia also found in the control population, it is very likely that these cases were identified antenatally at booking or later. The association of anaemia with sepsis was independent of mode of delivery and blood loss, supporting other findings that anaemia is an independent risk factor for sepsis, although the mechanism of this association is not yet understood.

Preterm birth and labour induction, which we found to be significantly associated with uncomplicated sepsis and severe sepsis, respectively, have also been previously cited.^18^ Induction of labour has become common in obstetric practice. In both the UK and the USA, approximately one in every five pregnant women has an induced labour,^46^,^47^ and the associated maternal and fetal risks for further intervention such as operative vaginal delivery and caesarean section are being increasingly recognised.^48^ A national study from the Netherlands found that induction of labour was associated with a 5.2 relative risk of sepsis.^18^ This same study found that preterm birth had a 10.3 relative risk of sepsis.^18^ Our results seem to support these findings. Limiting induction, therefore, to clearly indicated cases might be predicted to impact on the incidence of sepsis.

There were several limitations to this study. First, risk factors identified for severe sepsis could only be interpreted as exploratory because of a very small sample size; however, they largely reflected uncomplicated sepsis risk factors. Second, sources of infection and causative organisms were beyond the scope of the data used for this study. Third, we were unable to definitively ascertain the temporality of antibiotic usage in relation to mode of delivery or placental removal, but it is likely that women with sepsis mostly received treatment antibiotics for sepsis, whereas control women received prophylactic or early-stage treatment antibiotics. Similarly, we were unable to comment on the temporality of preterm birth and other factors, e.g. mode of delivery, as either a risk factor or an outcome of infectious morbidity, although our findings and those from other studies indicate that they are clearly associated with maternal sepsis. Lastly, because this study was geographically limited to one area of the UK, elements that are unique to this population, such as ethnicity, may not be representative of the wider UK population.

Despite these limitations, the findings from this study point to several implications for clinical practice. First, this study underscores the need for prophylactic antibiotics at caesarean section. The fact that there was much higher antibiotic usage among control women who had a caesarean section before discharge compared with women with sepsis, supports the amplitude of evidence demonstrating the efficacy of antibiotic prophylaxis at caesarean section. Second, the association we observed between operative vaginal delivery and maternal sepsis should encourage strict aseptic technique and infection control measures in clinical practice. As the prevalence of sepsis among women undergoing operative vaginal delivery was still very low in this study, a potential randomised clinical trial to investigate whether prophylactic antibiotics offer protection does not yet appear to be indicated. Further studies, however, should evaluate operative vaginal delivery as a potential risk factor for maternal sepsis. Lastly, obese pregnant women in the UK may benefit from additional monitoring during and after pregnancy to prevent critical infection leading to sepsis. Current Royal College of Obstetricians and Gynaecologists guidelines on the management of women with obesity in pregnancy do not suggest additional monitoring.^49^ This study and further research, however, may serve to emphasise the need for routine use of a modified early obstetric warning score (MEOWS) chart, particularly among obese and other high-risk women,^3^ to facilitate early detection of any obstetric complication.

## Conclusion

In this study we identify obesity as a significant risk factor for maternal sepsis, in addition to younger maternal age, operative vaginal delivery and other known risk factors such as multiparity, anaemia, labour induction, caesarean section and preterm birth. Women with sepsis who had a caesarean section were less likely than controls to receive antibiotics, highlighting the need for prophylaxis. The association we observed between operative vaginal delivery and maternal sepsis emphasises the importance of strict aseptic technique and infection control measures in clinical practice. The association between obesity and maternal sepsis is also of clinical significance given the concurrent increase in maternal obesity in the UK. This study, however, despite using a large population database, had limited power to investigate factors associated with severe maternal sepsis. We therefore suggest further research, including a national prospective cohort study, to comprehensively evaluate the magnitude of severe maternal sepsis morbidity, and to validate the risk factors identified in this study.
